# Cementless Fixation of Osteoporotic VCFs Using Titanium Mesh Implants (OsseoFix): Preliminary Results

**DOI:** 10.1155/2014/853897

**Published:** 2014-06-02

**Authors:** Anica Eschler, Stephan Albrecht Ender, Benjamin Ulmar, Philipp Herlyn, Thomas Mittlmeier, Georg Gradl

**Affiliations:** ^1^Department of Trauma, Hand and Reconstructive Surgery, University of Rostock Medical Center, Schillingallee 35, 18057 Rostock, Germany; ^2^Department of Orthopaedics and Orthopaedic Surgery, University Medicine Greifswald, Ferdinand-Sauerbruch Straße, 17475 Greifswald, Germany; ^3^Department of Trauma, Orthopedic and Reconstructive Surgery, Klinikum München Harlaching, Sanatoriumsplatz 2, 81545 München, Germany

## Abstract

*Introduction*. Vertebral compression fractures (VCFs) affect 20% of people over the age of 70 with increasing incidence. Kypho-/vertebroplasty as standard operative procedures are associated with limitations like cement leakage, limited reduction capabilities, and risk for adjacent fractures. To address these shortcomings, we introduce a new minimal invasive cementless VCF fixation technique. *Methods*. Four patients (72.3 years, range 70–76) with VCFs type AO/Müller A1.3 and concomitant osteoporosis were treated by minimal invasive transpedicular placement of two intervertebral mesh cages for fracture reduction and maintenance. Follow-up included functional/radiological assessment and clinical scores and averaged 27.7 months (24–28). *Results*. Endplate reduction was achieved in all cases (mean surgery time: 28.5 minutes). Kyphotic (KA) and Cobb angle revealed considerable improvements postoperatively (KA 14.5° to 10.7°/Cobb 10.1° to 8.3°). Slight loss of vertebral reduction (KA: 12.6°) and segment rekyphosis (Cobb: 10.7°) were observed for final follow-up. Pain improved from 8.8 to 2.8 (visual analogue scale). All cases showed signs of bony healing. No perioperative complications and no adjacent fractures occurred. *Conclusion*. Preliminary results in a small, selected patient collective indicate the ability of bony healing for osteoporotic VCFs. Cementless fixation using intravertebral titanium mesh cages revealed substantial pain relief, adequate reduction, and reduction maintenance without complications. Trial registration number is DRKS00005657, German Clinical Trials Register (DKRS).

## 1. Introduction

Due to the demographic changes, the European and North American populations are facing an increasing incidence of osteoporosis and therefore vertebral compression fractures (VCF) [1–3]. Especially in the elderly population with concomitant osteoporosis, VCFs frequently occur after minor trauma, initially often unnoticed since the symptoms are dismissed as signs of aging [4]. Preexisting kyphotic spine deformity bears a 6-fold risk of a second fracture in 20% of patients with previous VCF within a 1-year period [5–7]. This in turn is associated with a rise in back pain, reduced mobility, and reduced quality of life and finally a rise in morbidity and mortality rates due to decreased pulmonary function and abdominal constriction [8].

VCF treatment strategies combine nonoperative options, using pain medication and braces in first place whilst accepting a progress of spine deformity and minimal invasive operative procedures such as kyphoplasty and vertebroplasty which are long- and well-established methods [9–13]. Both, kyphoplasty and vertebroplasty involve the application of bone cement, regardless of the fracture severity. This seems problematic, since, even after minor traumata, VCFs are amenable for involvement of the dorsal wall [14]. When taking this into consideration, cement-based treatment algorithms for VCF care are likely to induce cement-associated complications like leakage in the disc space or epidural space in more severe fracture types like burst fractures with dorsal wall involvement [15–20].

Another problem with cement based treatment strategies is the nonabsorbality of the required PMMA cement leading to a life-long rigid implant which may support adjacent vertebral fractures [12, 21–23]. Until now it is not known as to whether the extensive filling of cement in a vertebral body has an adverse effect on bony healing [24].

For both treatment strategies, kypho- and vertebroplasty, procedural disadvantages as incomplete fracture reduction or loss of the restored height in the time frame between reduction with balloon deflation and cement application are reported [13, 25, 26].

In order to address those specific shortcomings, we treated osteoporotic VCFs by MIS (minimal invasive) internal fixation using two intracorporeal expandable titanium mesh cages for fracture reduction and maintenance of reduction, in off-label use without cement application. Thus, natural bony healing is not inhibited. First-step biomechanical trials performed by Upasani et al. [27] comparing the titanium mesh cages with cement application and the commonly used balloon kyphoplasty showed significant greater height maintenance and significant less cement amounts in the titanium mesh group. Second step biomechanical studies by Ghofrani et al. [21] compared the titanium mesh cages without cement application versus kyphoplasty in human spine cadavers and revealed similar results. One single third step studies showed that VCF stabilization combining cement application and the titanium mesh cages are effective in terms of fracture reduction with low complication rates in a clinical setting [14]. Up to now, there is very little knowledge of clinical applications of cementless VCF fixation using two titanium mesh cages. We hypothesized that this specific fixation technique for VCF stabilization (i) allows for adequate restoration of the vertebral height, (ii) shows maintenance of the reached restoration for at least two years, (iii) shows the same ability of pain reduction as extensively reported in the literature for solutions applying bone cement, and (iv) shows less adjacent fractures and complications as for solutions applying bone cement. This study indicates first preliminary results.

## 2. Material and Methods

Four vertebral bodies in four patients with acute VCF of the lumbar spine (AO type A1.3) were treated operatively from July 15 to November 15, 2011, and observed prospectively. Fracture fixation was done in percutaneous MIS technique using two titanium mesh implants (OsseoFix, Alphatec Spine, Carlsbad, California, US).

Mean age of patients (3 males, 1 female) was 72.3 years (range: 70–76 years). One patient suffered from more than three secondary diagnoses. Clinically, all patients presented with acute, painful back pain without posttraumatic new sensomotor deficits. By means of CT scanning, fracture type according to AO revealed type A1.3 fractures in all cases. Lumbar vertebra 1 was affected twice and 3 and 4 once each. Indication for surgery was endplate subsidence and low back pain not adequately responding to pain treatment WHO II for 3 weeks. Mean follow-up was 27.7 months (range: 24.8–28.8 months) without any patients lost for follow-up.

**Figure 1:**
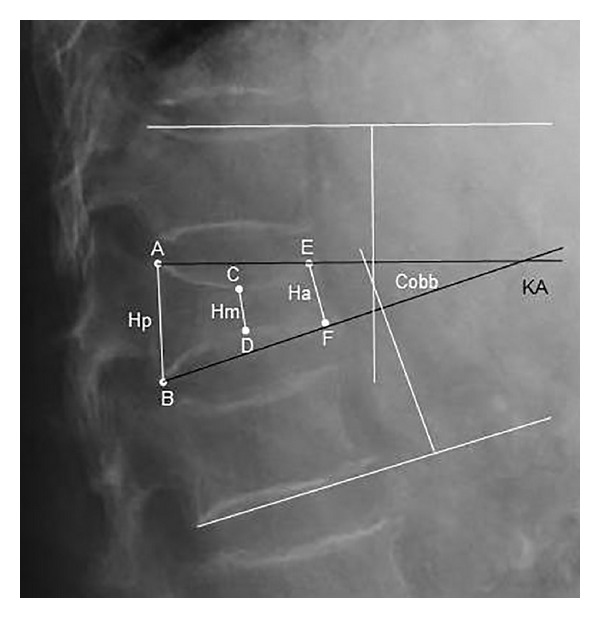
Radiological measurement by means of a fractured vertebra body of the thoracolumbar spine. Height in sagittal alignment is defined using 6 points: A and B are on the most dorsal-superior and dorsal-inferior endplate margins, E and F correspond to the most anterior-superior and anterior-inferior margins, and C and D are on the midpoint of a perpendicular line drawn from A to E and B to F on the superior and inferior vertebral endplates. Resulting Cobb and vertebra body kyphotic angle.

Radiological follow-up included standing anterior-posterior and lateral radiographs at four different time points: preoperatively, postoperatively, six months postoperatively, and for final follow-up (28 months postoperatively). Additional CT scans were available intraoperatively. Intra- and postoperative safety of the procedure as well as analysis of reduction quality was analyzed by fluoroscopic imaging. All radiographs were analyzed for changes in sagittal alignment referring to the posterior (Hp), middle (Hm), and anterior body height (Ha) according to six defined points on the fractured vertebra (A–F, Figure 1). The sagittal index (SI) as measurement of segmental kyphosis at the level of one spine segment was calculated from the posterior and anterior height. The vertebral body kyphotic angle (KA) was measured by means of the intersections connecting points AE and BF. The Cobb angle as measure for the sagittal alignment at the fracture level was calculated from the intersections of the tangents to the endplates of the corresponding vertebral bodies superior and inferior from the injured level.

Clinical follow-up included pain rate evaluation referring to a visual analogue scale (VAS) preoperatively, on the first and third postoperative days, six months and 28 months after surgery. A standardized subjective and functional assessment of the spine including the Roland Morris Questionnaire (RMQ) [28], Oswestry Disability Index (ODI) [29], range of motion (ROM) measurement, and testing of sensory and motor deficits were performed at six months and final follow-up. Results were given as mean ± SEM (range).

The study was approved by the local ethical committee under the document number A 2012-0004 and is in accordance with the declaration of Helsinki.

### 2.1. Surgical Technique

In order to reduce and stabilize the collapsed vertebral body, the patient was brought into a prone position and two up to 2 cm limited surgical incisions were made dorsolateral from the pedicle level after fluoroscopic identification of the injured vertebra. Using a targeting needle, two K-Wires were placed transpedicularly into the vertebral body under fluoroscopic control in two planes, followed by cannulated drilling of the pathway into the anterior third of the vertebral body. An implant delivery system helped to insert the two titanium mesh cages which had been selected regarding their proper size according to preoperative planning from preoperative CT scans. A mechanical actuation system was used to deploy the expandable cages in a controlled manner.

Postoperatively, full weight bearing was allowed immediately according to the patient's pain threshold.

## 3. Results

**Figure 2:**
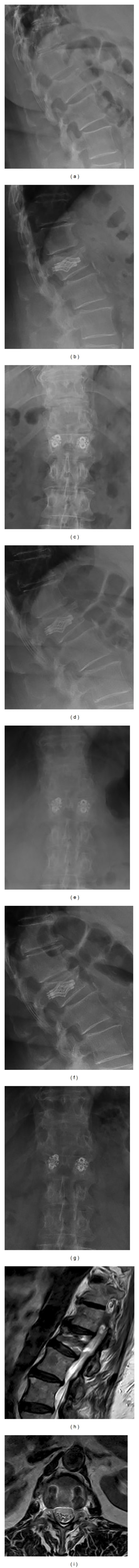
Radiographs pre- and postsurgery, six months and 28 months after surgery showing a lumbar VCF in an osteoporotic female patient and operative treatment using two titanium mesh cages placed in minimum-invasive technique transpedicularly. (a) preoperatively, ((b), (c)) postoperatively, ((d), (e)) for six months follow-up, ((f), (g)) for 28 months follow-up. ((h), (i)) MRI of the same patient six months after surgery showing bony healing of the vertebral fracture.

Stabilization and reduction of the compressed vertebral body was achieved in all four vertebrae without any intraoperative complications (Figure 2). Intraoperative fluoroscopic imaging proved correct cage positioning in all cases. Mean time for surgery was 28.5 ± 1.6 minutes (24–35 minutes). Mean time of postoperative hospitalisation came up to 5.0 ± 0.5 days (3–7 days); the preoperative hospitalisation period was 3.5 ± 1.0 days (0–7 days). All patients experienced an uncomplicated course of the disease.

### 3.1. Radiological Results

Intraoperative vertebral body reduction was achieved in all cases with respect to body angle and decrease of kyphotic angulation of the adjacent vertebra segments. Ha improved from 25.0 ± 2.6 mm (20–33 mm) preoperatively to 28.8 ± 1.8 mm (24–36 mm) postoperatively and stayed at this constant level for the whole follow-up period (27.3 ± 2.2 (23–36 mm) six months follow-up, 27.6 ± 2.1 (23–36 mm) 28 months follow-up). Hm did not show relevant changes and fortunately did not decrease as one might expect: 29.1 ± 1.4 mm (26–33 mm) preoperatively, 30.9 ± 0.9 mm (28–34 mm) postoperatively, 30.1 ± 1.0 mm (28–32 mm) for the six months follow-up, and 29.9 ± 0.8 mm (27–33 mm) for the 28 months follow-up. Hp was not expected and did not show any relevant changes since none of the fractures affected the dorsal wall (35.5 ± 0.7 mm (33–38 mm) preoperatively, 35.8 ± 0.6 mm (34–38 mm) postoperatively, 35.5 ± 0.6 mm (34–38 mm) after six months, and to 35.4 ± 0.6 mm (35–37 mm) after 28 months). These results imply that SI improved from 0.7 ± 0.1 (0.6–0.9) to 0.8 ± 0.0 (0.7–1.0) showing a tendency to less kyphosis of the vertebral body. In this small group of patients, we did not observe any rekyphosis in mean with SI 0.8 ± 0.1 (0.8–1.0) observed for the six months follow-up and SI 0.8 ± 0.1 (0.6–1.0) for the final follow-up.

**Figure 3:**
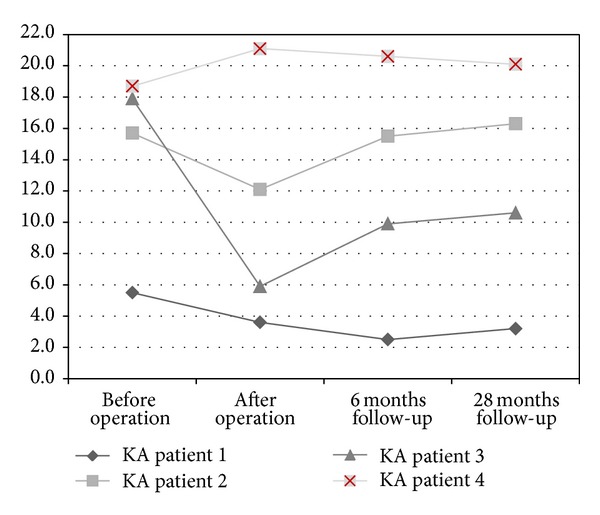
Radiographic evaluation of the KA angle for vertebral body reduction in sagittal alignment pre- and postoperatively, at six months and 28 months follow-up (in °).

Successful vertebral body reduction was proved by KA measurement revealing improvement from 14.5 ± 2.2° (6–19°) to 10.7 ± 3.0° (7–21°). During the whole follow-up period, a slight loss of reduction was observed (12.1 ± 2.9° (3–21°) six months follow-up, 12.6 ± 2.8° (3–20°) 28 months follow-up) KA (Figure 3).

**Figure 4:**
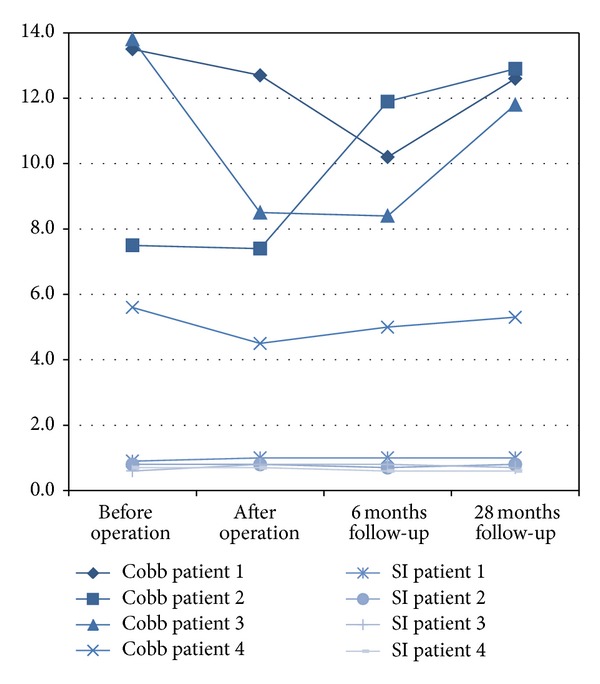
Radiographic evaluation for changes in the Cobb angle and SI index pre- and postoperatively, at six months and 28 months follow-up.

The Cobb angle as parameter for the sagittal alignment of the fracture level improved from 10.1 ± 1.8° (6–14°) to 8.3 ± 1.2° (5–13°) revealing less kyphosis (Figure 4). Six months after surgery 8.9 ± 1.1° (5–12°) angulation was measured and 28 months after surgery 10.7 ± 1.3° (5–13°) speaking in favor for a rekyphosis.

Follow-up X-rays after six months showed bony healing in all patients (Figures 2(d) and 2(e)). No implant dislocations were observed. During the whole study period, we did not observe any adjacent vertebral fracture.

### 3.2. Functional Results

**Figure 5:**
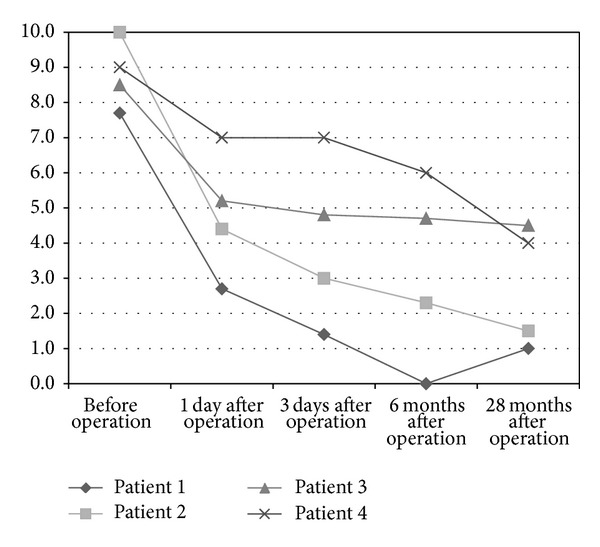
Pain level rated by VAS during the study period (0 = no pain, 10 = maximum pain).

Pain progress as rated by VAS accounted for 8.8 ± 3.5 (8–10) preoperatively and improved considerably to 4.8 ± 6.4 (3–7) on the first day after surgery. A further improvement was noticed on the third day after surgery (4.1 ± 9.3 (1–7)) and for the six months follow-up with (3.3 ± 10.5 (0–6)). In all cases except one, further decrease in pain rates was observed for final follow-up with a mean of 2.8 ± 7.5 (1–5) (Figure 5). All but one patient reported no limitations in their walking distance; one patient was limited to 100 meters pain-free mobilization. One patient took pain medication daily and another one sporadically.

The further clinical follow-up included the RMQ questionnaire as indicator for pain with activities of daily living. For the six months follow-up RMQ resulted in a total of 9.3 ± 2.6 points (3–18 points) which corresponds to minor restriction; for final follow-up, a total of 6.3 ± 1.9 points (2–11 points) were measured confirming further regredience of symptoms. Results for the ODI confirmed moderate disability with 26.3 ± 8.1% (10–53%) for six months follow-up with further declining tendency to 20.5 ± 3.6% (6–30%) for final follow-up.

Postural control analysis revealed normal harmonic gait patterns in 3 cases; 1 patient showed a walking insecurity to the same extend as preoperatively known from a dorsal foot flexion weakness.

Mean range of motion in thoracolumbar flexion resulted in 110 ± 3.8° (100–120°) and 17 ± 3.2° (10–25°) in extension six months after surgery. Only slight changes were seen for final follow-up with 108 ± 3.8° (100–120°) flexion and 23 ± 3.8° (10–30°) extension revealing almost unaffected back motion. Rotation was decreased to 18 ± 4.5° (10–30°) rightwards and 15 ± 5.8° (5–30°) leftwards (six months results, 26 ± 1.9° (20–30°) left-/rightwards for final follow-up). Lateral bending accounted for 23 ± 6.4° (10–40°) rightwards and 20 ± 7.7° (10–40°) leftwards (six months results, 29 ± 3.1° (20–40°) left-/rightwards for final follow-up). The minimum distance between fingertip and the floor was 15.7 ± 7.4 cm (0–35 cm) in mean for the six months follow-up and 13.8 ± 4.4 cm (0–25 cm) for the 24 months follow-up.

## 4. Discussion

In principal, this study shows that cementless fixation of osteoporotic VCFs using expandable titanium mesh cages allows for intraoperative fracture reduction, short-term maintenance of reduction, and a considerable pain reduction in a limited number of patients. Long-term maintenance of reduction, however, was only partly achieved with a tendency of rekyphosis. Furthermore, radiographic imaging revealed that bony healing of osteoporotic VCFs in the lumbar spine is possible.

As compared to kypho- and vertebroplasty as the standard VCF treatment methods, our radiological results with improvements of 14.5° to 10.7° KA and 10.1° to 8.3° Cobb angle show similar data in terms of fracture reduction and maintenance. Kyphoplasty restores vertebral height by means of a balloon inflation; however, it does not ideally restore the KA angle in 34% of cases [30]. Vertebroplasty, which restores height without any intrinsic mechanical reduction devices (reduction relying on the patient positioning), fails reduction even more frequently [26, 31]. In both methods, a secondary loss of height in 18–63% is reported [32, 33]. We did not observe a loss of vertebral body height for one of the patients during the 28 months follow-up, still the vertebra segment in a certain degree adapted to a kyphosis which is likely related to a damage of the vertebral discs.

Our clinical outcome variables showed an average reduction in pain intensity based on the VAS, from 8.8 to 2.8 for final follow-up thus presenting comparable and partly preferable results to kypho-/vertebroplasty procedures (8.7–7.3 to 5.4–2.2) [34–36]. Astonishingly a constant pain relief was observed during the whole study period with still declining measures after 28 months. Analogue ODI functional results improved constantly. A meta-analysis of 69 clinical trials showed comparable results for pain relief and function for both vertebro- and kyphoplasty [37, 38]. These findings speak in favor of a cement-independent mechanism of pain reduction.

In fact, kyphoplasty and vertebroplasty are both based on PMMA cement application for fracture stabilization. Fracture stabilization using the two titanium mesh cages relies on a mechanical actuation system solely, in absence of cement. This leads to bony healing of osteoporotic VCFs in a small patient collective. Bony consolidation around a cement block may occur; however, it might be more likely that the cement had adverse effects on the bony consolidation process [17, 39]. An experimental study showed no negative nonunion rates for osteoporotic fracture healing (conservative) but nevertheless observed a decreased quantity and quality of callus mineralization [40]. Another problem following cement application is the changed biomechanical behavior (increased stiffness) of the vertebral body which in turn promotes other complications as adjacent fracture development [5, 6, 21–23, 41]. Adjacent vertebral fractures are the most common complications reported in the literature with up to 8% after vertebroplasty and 8 to 26% after kyphoplasty [19, 42–44]. We did not observe any adjacent fractures in this small patient collective. As for intraoperative complications after kypho-/vertebroplasty, leakage into the epidural space causing neurologic symptoms and cement leakage into the venous system due to high pressure forces used whilst application leading to pulmonary embolism are frequently described [16–18, 45, 46]. Uncontrolled 4 to 13% leakage rates for kyphoplasty and 20 to 70% for vertebroplasty were described in the literature [47, 48].

With the cementless application of the expandable titanium mesh cage, we did not see any of these complications leading to favorable results in complication rates when compared to standard methods [19, 20].

There is only one clinical trial published using the titanium mesh cages, still in combination with PMMA cement, revealing similar improvements in pain (VAS 7.7 to 1.4) and ODI (71% to 30%) and reduction (Cobb 11.7° to 10.4°) in a 12 months follow-up of 32 osteoporotic VCFs and treatment [14]. The same technique used without cement in off-label use is not reported in the literature so far.

In terms of operation time, this new technique glares with 28.5 min in this limited number of patients, which is comparable to kypho-/vertebroplasty techniques (24–120 min) [16, 49–53]. Above-mentioned study using titanium mesh cages in combination with PMMA cement reported 55 min operation time which again speaks in favor for our technique with similar outcome parameters in absence of cement-associated risks.

There might be another advantage for the titanium mesh technique in terms of the level of patient's and surgeon's radiation exposure which is problematic in kypho-/vertebroplasty [54]. Radiation exposure was not addressed in this study, but still is suggestively lower since intense fluoroscopic controls whilst cement application are not necessary. Anyhow, the main limitation of this study is the small preliminary patient collective which limits comparability to larger case serious in the literature. Those are needed to show if clinical results are comparable to standard techniques and complication counts can effectively be reduced.

## 5. Conclusion

Preliminary results in a limited number of patients indicate that cementless fixation of VCFs employing titanium mesh cages is able to restore vertebral geometry and is very effective in pain reduction. Maintenance of reduction is less effective, however, comparable to vertebro/kyphoplasty. Radiologic imaging led to the assumption of bony healing in all patients which may be beneficial for the biomechanical properties of the spinal rod. Cement-associated complications are omitted. Appraisal of all results should clearly implicate the main limitation of the study, the small patient collective which therefore makes them generally less conclusive. Further prospective randomized investigations are needed to demonstrate a putative superiority.
